# The prevalence, awareness, treatment, and control of dyslipidemia in northeast China: a population-based cross-sectional survey

**DOI:** 10.1186/s12944-017-0453-2

**Published:** 2017-03-23

**Authors:** Fu-Liang Zhang, Ying-Qi Xing, Yan-Hua Wu, Hao-Yuan Liu, Yun Luo, Ming-Shuo Sun, Zhen-Ni Guo, Yi Yang

**Affiliations:** 1 Stroke Center, Department of Neurology, the First Hospital of Jilin University, Xinmin Street 71#, 130021 Changchun, China; 2 Neuroscience Center, Department of Neurology, the First Hospital of Jilin University, Xinmin Street 71#, 130021 Chang Chun, China; 3 Division of Clinical Research, the First Hospital of Jilin University, Changchun, China

**Keywords:** Dyslipidemia, Prevalence, Awareness rate, Treatment rate, Control rate, Epidemiology, Northeast China

## Abstract

**Background:**

Dyslipidemia is an important independent modifiable risk factor for cardiovascular disease. The aim of this study was to explore the current prevalence, awareness, treatment and control of dyslipidemia and its associated influence factors in northeast China.

**Methods:**

In this population-based cross-sectional study, we adopted a multi-stage, stratified sampling method to obtain a representative sample of 4052 permanent residents aged 40 years and over from different urban and rural regions in Dehui City of Jilin Province. All subjects completed a questionnaire and were examined for risk factors. Continuous data were presented as means ± standard deviations (SD) and compared using the Student’s *t*-test. Categorical variables were presented as proportions and compared using the Rao-Scott-*χ* ^2^ test in different subgroups. The associated influence factors for the prevalence, awareness, treatment and control of dyslipidemia were evaluated through multivariate logistic regression.

**Results:**

The prevalence of dyslipidemia was 62.1% overall, with 33.5, 43.9, 0.6, and 8.8% for high total cholesterol, triglyceride, low-density lipoprotein cholesterol, and low high-density lipoprotein cholesterol, respectively. Among those with dyslipidemia, the proportion of subjects who were aware, treated, and controlled was 14.4, 33.9, and 19.9%, respectively. Overweight or obesity (OR = 2.156; 95% CI: 1.863, 2.533), hypertension (OR = 1.643; 95% CI: 1.425, 1.893), or diabetes mellitus (OR = 2.173; 95% CI: 1.661, 2.844) increased the prevalence of dyslipidemia, also these participants were more likely to be aware of their condition, however, this did not increase the likelihood of treatment and control. Living in urban areas and higher education level also increased the awareness of dyslipidemia. Personal history of coronary heart disease was the strongest influence factors associated with better awareness, treatment and control of dyslipidemia. Overweight or obesity (OR = 0.404; 95% CI: 0.235, 0.695) and lack of exercise (OR = 0.423; 95% CI: 0.215, 0.830) were associated with poor control of dyslipidemia.

**Conclusion:**

The prevalence of dyslipidemia among adults aged 40 years and over in northeast China was high, however, the awareness, treatment, and control of dyslipidemia was measured at far from desirable levels. Renewed efforts taking influence factors into account are needed to improve the current unsatisfactory condition.

## Background

Coronary heart disease and cerebrovascular disease, as two major types of cardiovascular disease (CVD), were ranked as the top two caused of premature death and disability-adjusted life-years (DALYs) worldwide [1, 2], and contributed considerably to the increasing healthcare expenditures, especially in low- and middle-income countries, as well as in China [3, 4]. With the rapid economic expansion and increasing average life expectancy, CVD has become the leading cause of death in China, and its morbidity and mortality has been increasing since the late 1980s. Currently, CVD accounts for up to 40% of all deaths in both urban and rural populations in China [5].

On a more positive note, a considerable proportion of this morbidity and mortality could be prevented through early intervention of cardiovascular risk factors, such as low physical activity, tobacco use, dyslipidemia, hypertension, diabetes, etc.[6, 7], in which dyslipidemia plays an important role in the formation and development of atherosclerosis, and its association with risk of CVD is indisputable [8–10]. Therefore, early screening and effective lipid management may substantially reduce the burden of CVD and provide great social value [3].

With the improved standard of living, unhealthy dietary patterns and sedentary lifestyles have increased, and the age-standardized prevalence rate of dyslipidemia has been gradually increasing [3, 11, 12]. Moreover, this rate varies by region due to different climatic environments, diets, and lifestyles [13]. The latest data from Jilin Province in northeast China reported that the awareness, treatment, and control rate of dyslipidemia were 11.6, 8.4, and 34.8%, respectively, in 2012 [14]. However, this data did not contain the prevalence rates of dyslipidemia or details regarding the subtypes of lipid disorders (high serum total cholesterol, triglycerides, low-density lipoprotein cholesterol, and low high-density lipoprotein cholesterol). The 2015–16 Stroke Screening and Prevention Program of the National Health and Family Planning Commission of China Study includes these data and remains the latest cross-sectional survey in northeast China. Based on this survey database, we analyzed the current prevalence, awareness, treatment, and control of dyslipidemia and its associated influence factors in Jilin Province, northeast China.

## Methods

### Data source and study population

This survey was conducted from January to March 2016 as part of the Stroke Screening and Prevention Program of the National Health and Family Planning Commission of China [15]. It was a population-based cross-sectional study designed to provide timely and reliable data for the prevalence of stroke and associated factors in adults over 40 years of age in northeast China, and it was supervised by the Chinese National Center for Stroke Care Quality Control and Management. This survey adopts the multistage stratified random cluster sampling method to select representative samples of the general population over 40 years who had lived in Dehui City for more than 6 months and voluntarily participated in the survey. In the first stage, 30 villages and 10 towns were selected from 308 villages (rural) and 14 towns (urban), respectively, in Dehui city. In the second stage, cluster sampling was used in the areas selected above. The study was approved by the Human Ethics and Research Ethics committees of the First Hospital of Jilin University (Approval Number: 2015-R-250). Written informed consent was obtained from all participants in the survey.

### Sampling size

The targeted population for the Stroke Screening and Prevention Program of the National Health and Family Planning Commission of China 2015–16 were adults aged 40 years and over, and there were 335,490 residents over 40 years in Dehui City, according to the main data bulletin of the 6th National Population Census in 2010 (announced by the Statistical Bureau of Jilin Province). The expected sample size was 3355, which was 1% of the targeted population. Non-response rate was estimated as 20%, so the planned sample size was 4026; a final number of 4100 residents participated in the survey. For the purpose of the present analysis, 48 subjects were excluded due to missing values. Finally, a total of 4052 people were included in the present analysis.

### Data collection and measurement

All participants completed a structured pre-coded questionnaire designed by the National Health Development Planning Commission for Stroke Screening and Prevention Engineering, including demographic details (e.g., gender, age, residence, and education level), personal lifestyle (e.g., smoking, drinking, exercise habits, and diet structure), personal and family medical history of stroke and chronic diseases (i.e., hypertension, diabetes mellitus, dyslipidemia, and atrial fibrillation), and anthropometric measurements (e.g., height, weight, resting blood pressure, neck, waist, and hip circumference). Height and weight were measured according to standardized protocol and techniques, with the participants wearing clothes but no shoes. Blood pressure was measured by trained professionals using an electronic sphygmomanometer (OMRON HEM-7200), and each participant was measured twice, resting for at least 20 min before measurements were taken. We took the average of these readings. In addition, a blood sample was drawn from the participant’s antecubital vein for measuring fasting blood glucose (FBG), total cholesterol (TC), triglycerides (TGs), low-density lipoprotein cholesterol (LDL-C), and high-density lipoprotein cholesterol (HDL-C), and the sample was uniformly measured by the Changchun Kingmed Center for Clinical Laboratory Co., Ltd. All participants underwent electrocardiograms for the detection of atrial fibrillation.

### Assessment criteria

Dyslipidemia [16] was defined as using an antilipidemic medication or having one or more of the following in the field survey: TC ≥ 5.18 mmol/L, TGs ≥ 1.70 mmol/L, LDL-C ≥ 3.37 mmol/L, and. HDL-C < 1.04 mmol/L.

Dyslipidemia awareness was defined as self-report of any previous diagnosis of dyslipidemia by a healthcare professional. The treatment rate of dyslipidemia was defined as the self-reported use of lipid-lowering drugs among participants who were aware of dyslipidemia. The control rate of dyslipidemia refers to the proportion among those treated for dyslipidemia who reach the lipid standard: TG < 1.70 mmol/L, TC < 5.18 mmol/L, HDL-C ≥ 1.04 mmol/L, and LDL-C < 3.37 mmol/L.

### Statistical analysis

Complex weighted computation was used to make the sample more accurately representative of the population in Dehui City of the Jilin Province by post-stratification adjustment according to the following factors: age, residence, and gender groups, according to the standard population in the 6th national general investigation in Dehui City of the Jilin Province. Continuous data are presented as means ± standard deviations (SD) and compared using the Student’s *t*-test. Categorical variables were presented as proportions and compared using the Rao-Scott-*χ* ^2^ test in different subgroups. Finally, the associated influence factors for the prevalence, awareness, treatment, and control of dyslipidemia were analyzed through multivariate logistic regression. All statistical analyses were performed using the complex samples function of IBM SPSS 17.0 (SPSS Inc., New York, NY, USA). Statistical significance was set at *p* < 0.05.

## Results

### Characteristics of the study sample

The study’s 4052 participants included 1619 men (mean age was 55.72 ± 9.43 years) and 2433 women (mean age was 54.27 ± 9.17 years). The number of cases represented by urban participants was 2067 (51.0%) and that of rural participants was 1985 (49.0%).

Demographic and clinical characteristics of participants enrolled in this cross-sectional population-based survey according to dyslipidemia status are shown in Table 1. Compared with the non-dyslipidemia group, we found that the dyslipidemia group had a higher prevalence of hypertension, diabetes mellitus, overweight or obesity, lack of exercise, personal and family history of stroke and coronary heart disease, family history of dyslipidemia, and central obesity (*p* < 0.001, except *p* < 0.05 for family history of stroke). In addition to this, the dyslipidemia group was usually older and had higher systolic blood pressure and diastolic blood pressure, higher fasting blood-glucose, and higher neck, waist, and hip circumferences than those of the non-dyslipidemia group (all *p* < 0.001).

**Table 1:** Characteristics of participants according to dyslipidemia status

Characteristics	Dyslipidemia (*n* = 2570)	Normal (*n* = 1482)	t	*p* value
Age (years)	55.52 ± 9.15	53.68 ± 9.45	6.104	<0.001
Gender (Male)	1027 (40.0)	592 (39.9)		0.992
Residence				0.037
Urban, n (%)	1343 (52.3)	724 (48.9)		
Rural, n (%)	1227 (47.7)	758 (51.1)		
Education, n (%)				0.163
Primary school and below	934 (36.3)	512 (34.5)		
Junior middle school	1070 (41.6)	626 (42.2)		
Senior middle school	341 (13.3)	196 (13.2)		
College and above	225 (8.8)	148 (10.0)		
^a^Hypertension, n (%)	1668 (64.9)	667 (45.0)		<0.001
^b^Diabetes mellitus, n (%)	366 (14.2)	75 (5.1)		<0.001
^c^Overweight or obesity, n (%)	962 (37.4)	277 (18.7)		<0.001
^d^Lack of exercise, n (%)	623 (24.2)	279 (18.8)		<0.001
^e^Personal history of stroke, n (%)	219 (8.5)	73 (4.9)		<0.001
^f^Family history of stroke, n (%)	921 (35.8)	476 (32.1)		0.016
^g^Personal history of coronary heart disease, n (%)	207 (8.1)	65 (4.4)		<0.001
^h^Family history of coronary heart disease, n (%)	694 (27.0)	317 (21.4)		<0.001
^i^Family history of dyslipidemia, n (%)	498 (19.4)	126 (8.5)		<0.001
BMI (kg/m^2^), n (%)				<0.001
BMI < 18.0	25 (1.0)	39 (2.6)		
18 ≤ BMI < 24.0	944 (36.7)	884 (59.6)		
24 ≤ BMI < 26.0	639 (24.9)	282 (19.0)		
26 ≤ BMI < 28.0	497 (19.3)	154 (10.4)		
BMI ≥ 28.0	465 (18.1)	123 (8.3)		
^j^Central obesity, n (%)	1516 (59.0)	528 (35.6)		<0.001
SBP (mmHg)	144.16 ± 21.61	135.58 ± 20.73	12.485	<0.001
DBP (mmHg)	90.46 ± 11.91	85.86 ± 11.03	12.416	<0.001
TG CHOHDLLDL (mmol/L)				
TC	5.77 ± 1.21	4.75 ± 0.68	34.294	<0.001
TG	2.61 ± 2.01	1.12 ± 0.31	36.703	<0.001
LDL-C	2.16 ± 0.77	2.09 ± 0.85	2.2652	0.008
HDL-C	1.22 ± 0.26	1.31 ± 0.20	−11.132	<0.001
FBG (mmol/L)	5.52 ± 1.88	4.98 ± 1.08	11.640	<0.001
Neck circumference (cm)	34.71 ± 3.37	33.40 ± 2.94	12.936	<0.001
Waist circumference (cm)	87.51 ± 8.86	82.27 ± 8.99	18.035	<0.001
Hip circumference (cm)	97.77 ± 8.25	93.50 ± 8.36	15.737	<0.001
Fruit consumption, n (%)				0.998
≥ 5d/w	2273 (88.4)	1307 (88.2)		
3-4d/w	223 (8.7)	136 (9.2)		
≤ 2d/w	74 (2.9)	39 (2.6)		
^k^Smoker				0.231
Yes	1304 (50.7)	723 (48.8)		
No	1266 (49.3)	759 (51.2)		
Alcohol consumption, n (%)				0.887
Never	1878 (73.1)	1100 (74.2)		
Moderate drinking	399 (15.5)	242 (16.3)		
Excessive drinking	293 (11.4)	140 (9.4)		

*Abbreviations*: *BMI* body mass index, *SBP* systolic blood pressure, *DBP* diastolic blood pressure, *TC* total cholesterol, *TG* triglyceride, *LDL-C* low-density lipoprotein cholesterol, *HDL-C* high-density lipoprotein cholesterol; FBG, fasting blood-glucose

^a^Hypertension was defined as self-reported history and/or the use of antihypertensive medication in the past 2 weeks, or the average of two times resting systolic blood pressure (SBP) ≥ 140 mmHg and/or diastolic blood pressure (DBP) ≥ 90 mmHg in the field survey

^b^Diabetes mellitus was defined as the use of insulin and/or oral hypoglycemic medications or a self-reported history of diabetes or FBG ≥ 7.0 mmol/L in the field survey

^c^Overweight or obesity was defined as BMI ≥ 26 kg/m^2^. Body mass index (BMI) was calculated as weight (kg) divided by height squared (m^2^)

^d^Lack of exercise was defined as physical exercise < 3 times a week for < 30 min each time, and this included industrial and agricultural labor

^e^Respondents with a history of stroke were asked to provide their outpatient or inpatient medical records, and the diagnostic criteria met the WHO criteria for stroke

^f^Family history of stroke was restricted to immediate family members

^g^Respondents with a history of coronary heart disease were asked to provide their outpatient or inpatient medical records, and the diagnostic criteria met the WHO criteria for coronary heart disease

^h^Family history of coronary heart disease was restricted to immediate family members

^i^Family history of dyslipidemia was restricted to immediate family members

^j^Central Obesity was defined by waist circumference >90 cm for male and >80 cm for female

^k^A smoker was defined as one who reported having smoked one or more cigarettes or was passively exposed to tobacco smoke every day in general for more than 6 consecutive months

### Prevalence of different types of dyslipidemia

As shown in Table 2, the overall adjusted prevalence of dyslipidemia was 62.1% (95% CI: 60.3–63.9%). It first increased, and then decreased with age (*p* < 0.05), and the peak prevalence appeared in the 60–69 year age group, and the same trend occurred in high total cholesterol measurements (*p* < 0.001). The prevalence of dyslipidemia showed no statistically significant difference between males and females (61.3% vs. 62.9%, *p* = 0.383), but that of low high-density lipoprotein cholesterol was significantly higher in males than females (11.1% vs. 6.4%, *p* < 0.001), yet the opposite was true for high total cholesterol (31.6% vs. 35.5%, *p* = 0.031). The adjusted prevalence rates of high total cholesterol, triglycerides, low-density lipoprotein, and low high-density lipoprotein were 33.5, 43.9, 0.6, and 8.8%, respectively. Compared with the rural areas, participants in urban areas had a higher prevalence of high triglyceride, low high-density lipoprotein, and dyslipidemia (all *p* < 0.001). The prevalence of high total cholesterol decreased with education level (*p* < 0.001); however, that of low high-density lipoprotein showed the opposite trend (*p* < 0.001). With regard to body weight, the prevalence rates of dyslipidemia, high total cholesterol, triglycerides, low-density lipoprotein, and low high-density lipoprotein cholesterol were significant higher among those overweight or obesity than in others (*p* < 0.001, except *p* < 0.05 for high low-density lipoprotein). The prevalence of any types of dyslipidemia was not significantly associated with smoking or alcohol consumption status (all *p* > 0.05). In addition, subjects who have personal or family history of stroke and coronary heart disease, or family history of dyslipidemia had higher prevalence of dyslipidemia than those without any history of these conditions (all *p* < 0.05); and participants with family history of stroke were more likely to suffer from high low-density lipoprotein cholesterol compared to those without family history of stroke (1.5% vs. 0.1%, *p* < 0.001). Whereas, participants with family history of coronary heart disease or dyslipidemia had higher levels of prevalence of high total cholesterol and triglyceride as against those without any history of these conditions (*p* < 0.05).

**Table 2:** Prevalence of different types of dyslipidemia in northeast China

Category	Subcategory	H-TC	H-TG	H-LDL	L-HDL	Dyslipidemia
Total		33.5(31.8–35.3)	43.9(42.0–45.7)	0.6(0.3–1.0)	8.8(7.8–9.8)	62.1(60.3–63.9)
Age	40–49	27.3(24.4–30.4)	44.2(40.9–47.5)	0.6(0.2–1.6)	9.2(7.5–11.2)	58.6(55.3–61.8)
	50–59	37.3(34.5–40.2)	45.5(42.6–48.5)	0.5(0.2–1.4)	8.6(7.1–10.3)	64.4(61.4–67.2)
	60–69	38.5(35.3–41.8)	44.3(41.0–47.6)	0.3(0.1–1.1)	7.9(6.4–9.7)	65.7(62.4–68.9)
	70–	38.2(32.1–44.7)	37.2(31.3–43.6)	0.8(0.2–3.4)	9.1(6.3–13.0)	63.3(56.8–69.3)
	*p* value	<0.001	0.092	0.863	0.792	0.014
Gender	Male	31.6(28.9–34.3)	42.2(39.4–45.1)	0.7(0.4–1.5)	11.1(9.5–12.9)	61.3(58.5–64.1)
	Female	35.5(33.2–37.8)	45.6(43.2–47.9)	0.4(0.1–0.9)	6.4(5.4–7.6)	62.9(60.6–65.2)
	*p* value	0.031	0.075	0.236	<0.001	0.383
Residence	Urban	28.8(26.8–30.9)	49.6(47.3–51.8)	0.2(0.1–0.7)	15.6(14.0–17.4)	66.4(64.2–68.4)
	Rural	35.1(32.9–37.4)	42.0(39.6–44.3)	0.7(0.4–1.2)	6.5(5.4–7.8)	60.7(58.3–63.0)
	*p* value	<0.001	<0.001	0.089	<0.001	<0.001
Education	Primary school and below	36.8(34.1–39.6)	43.7(40.9–46.6)	0.6(0.3–1.2)	6.9(5.6–8.6)	62.9(60.0–65.6)
	Junior middle school	31.3(28.6–34.2)	43.4(40.5–46.4)	0.6(0.2–1.6)	9.6(8.1–11.3)	60.5(57.5–63.4)
	Senior middle school	30.7(26.3–35.5)	44.7(39.9–49.7)	0.2(0.0–1.7)	12.6(9.6–16.3)	64.2(59.3–68.9)
	College and above	23.6(19.1–28.9)	47.7(42.1–53.4)	0.4(0.1–3.1)	13.6(10.3–17.8)	63.9(58.5–69.0)
	*p* value	<0.001	0.685	0.878	<0.001	0.391
Lack of exercise	Yes	33.9(30.1–38.0)	49.9(45.8–54.0)	0.1(0.0–0.4)	12.3(10.1–14.8)	67.4(63.3–71.2)
	No	33.4(31.5–35.4)	42.6(40.5–44.6)	0.7(0.4–1.2)	8.0(7.0–9.2)	61.0(58.9–63.0)
	*p* value	0.839	0.002	0.003	<0.001	0.006
Smoking	Yes	34.1(31.7–36.5)	44.4(41.9–46.9)	0.5(0.3–1.1)	8.4(7.1–9.8)	62.4(59.9–64.8)
	No	32.7(30.1–35.3)	43.1(40.4–45.8)	0.6(0.3–1.5)	9.4(8.0–11.1)	61.7(58.9–64.3)
	*p* value	0.433	0.478	0.787	0.314	0.691
Overweight or obesity	Yes	38.5(35.3–41.8)	62.0(58.7–65.2)	1.1(0.5–2.4)	13.6(11.4–16.0)	77.2(74.3–79.9)
	No	31.3(29.2–33.4)	35.7(33.6–37.9)	0.3(0.1–0.7)	6.6(5.7–7.7)	55.3(53.0–63.7)
	*p* value	<0.001	<0.001	0.012	<0.001	<0.001
Alcohol consumption	Never	33.7(31.7–35.8)	43.9(41.8–46.0)	0.5(0.2–1.0)	8.7(7.6–9.9)	62.0(59.9–64.1)
	Moderate drinking	31.4(27.2–35.9)	43.5(39.0–48.2)	0.7(0.2–2.4)	9.4(7.1–12.4)	61.2(56.6–65.7)
	Excessive drinking	35.7(30.6–41.2)	44.4(38.9–50.0)	0.6(0.2–2.6)	8.4(6.0–11.6)	64.1(58.5–69.4)
	*p* value	0.438	0.973	0.859	0.814	0.712
Personal history of stroke	Yes	38.5(32.2–45.3)	50.9(44.1–57.6)	0.1(0.0–0.8)	11.5(7.9–16.5)	73.5(67.1–79.0)
	No	33.1(31.3–35.0)	43.3(41.4–45.3)	0.6(0.3–1.1)	8.6(7.6–9.6)	61.2(59.3–63.1)
	*p* value	0.109	0.035	0.078	0.137	<0.001
Family history of stroke	Yes	35.1(32.1–38.2)	46.1(42.9–49.3)	1.5(0.8–2.7)	10.0(8.2–12.1)	66.0(62.9–68.9)
	No	32.7(30.6–34.9)	42.8(40.5–45.0)	0.1(0.0–0.4)	8.2(7.1–9.4)	60.2(57.9–62.4)
	*p* value	0.221	0.098	<0.001	0.099	0.003
Personal history of coronary heart disease	Yes	27.8(22.3–34.1)	50.7(43.6–57.7)	0.3(0.0–2.4)	12.1(8.4–17.2)	69.6(62.4–76.0)
	No	33.9(32.1–35.8)	43.4(41.5–45.3)	0.6(0.3–1.0)	8.5(7.6–9.6)	61.6(59.7–63.5)
	*p* value	0.066	0.051	0.618	0.075	0.035
Family history of coronary heart disease	Yes	38.2(34.6–41.9)	47.2(43.4–50.9)	0.8(0.3–2.3)	8.8(6.9–11.2)	67.6(64.0–71.1)
	No	32.0(30.1–34.1)	42.8(40.7–45.0)	0.5(0.2–0.9)	8.8(7.7–10.0)	60.4(58.2–62.5)
	*p* value	0.003	0.049	0.427	0.938	0.001
Family history of dyslipidemia	Yes	47.3(42.8–51.8)	57.5(53.0–61.9)	1.2(0.5–3.1)	10.0(7.6–13.1)	79.8(75.9–83.1)
	No	30.3(28.4–32.2)	40.7(38.7–42.7)	0.4(0.2–0.8)	8.5(7.5–9.6)	57.9(55.9–60.0)
	*p* value	<0.001	<0.001	0.054	0.290	<0.001

*Abbreviations*: *H-TC* high total cholesterol, *H-TG* high triglyceride, *H-LDL* high low-density lipoprotein cholesterol, *L-HDL* low high-density lipoprotein cholesterol

### Awareness, treatment, and control of dyslipidemia

The overall awareness, treatment, and control rates of dyslipidemia were 14.4% (95% CI: 12.9–16.0%), 33.9% (95% CI: 28.5–39.8%), and 19.9% (95% CI: 15.5–25.2%), respectively (shown in Table 3).

**Table 3:** Awareness, treatment and control of dyslipidemia

Category	Subcategory	Dyslipidemia		
		Awareness	Treatment	Control
Total		14.4(12.9–16.0)	33.9(28.5–39.8)	19.9(15.5–25.2)
Age	40–49	11.6(9.2–14.6)	35.3(24.4–48.0)	17.7(10.0–29.2)
	50–59	13.7(11.5–16.3)	29.8(22.2–38.7)	14.6(9.0–22.7)
	60–69	18.5(15.6–21.8)	34.5(26.3–43.8)	21.8(15.0–30.6)
	70–	18.9(13.3–26.0)	38.7(22.7–57.6)	33.1(18.3–52.1)
	*p* value	0.007	0.763	0.123
Gender	Male	15.2(12.9–17.9)	31.2(23.4–40.1)	22.2(15.5–30.6)
	Female	13.5(11.7–15.6)	36.9(29.8–44.6)	17.4(12.4–23.9)
	*p* value	0.287	0.320	0.314
Residence	Urban	19.7(17.5–22.0)	30.1(24.6–36.2)	15.9(11.7–21.2)
	Rural	12.4(10.5–14.5)	36.1(28.3–44.7)	22.3(15.9–30.2)
	*p* value	<0.001	0.237	0.130
Education	Primary school and below	12.0(9.9–14.5)	28.9(20.7–38.8)	18.8(11.8–28.6)
	Junior middle school	14.4(12.0–17.2)	39.6(30.5–49.4)	21.3(14.4–30.4)
	Senior middle school	22.3(17.5–27.8)	37.4(25.2–51.6)	17.0(9.6–28.4)
	College and above	23.2(17.9–29.6)	26.1(15.4–40.7)	23.4(13.1–38.3)
	*p* value	<0.001	0.225	0.847
Lack of exercise	Yes	19.8(16.1–24.1)	27.0(18.5–37.5)	10.2(5.2–19.0)
	No	13.1(11.5–14.8)	36.4(29.9–43.4)	23.4(17.9–30.0)
	*p* value	0.001	0.128	0.013
Smoking	Yes	13.6(11.7–15.9)	31.2(23.9–39.5)	18.8(12.9–26.6)
	No	15.5(13.4–18.0)	37.8(30.2–46.0)	21.4(15.6–28.8)
	*p* value	0.236	0.245	0.589
Overweight or obesity	Yes	20.8(17.9–24.0)	32.1(24.9–40.3)	13.7(8.8–20.7)
	No	10.3(8.8–12.1)	36.2(28.4–44.7)	27.7(20.6–36.1)
	*p* value	<0.001	0.476	0.006
Alcohol consumption	Never	14.4(12.6–16.3)	37.0(30.6–44.0)	19.7(14.7–25.8)
	Moderate drinking	12.1(8.8–16.5)	30.2(16.9–48.0)	27.2(14.8–44.4)
	Excessive drinking	17.7(13.5–23.0)	24.1(13.6–39.0)	13.3(5.6–28.6)
	*p* value	0.185	0.282	0.329
Personal history of stroke	Yes	27.4(21.1–34.9)	43.3(29.7–58.0)	18.3(9.2–33.1)
	No	13.1(11.6–14.8)	32.1(26.3–38.5)	20.2(15.4–26.0)
	*p* value	<0.001	0.147	0.777
Family history of stroke	Yes	15.1(12.6–17.9)	39.6(30.6–49.4)	20.1(13.4–28.8)
	No	14.0(12.1–16.0)	30.5(24.1–37.8)	19.8(14.3–26.7)
	*p* value	0.505	0.122	0.960
Personal history of coronary heart disease	Yes	33.2(26.0–41.2)	44.8(31.7–58.7)	29.4(18.2–43.9)
	No	12.9(11.4–14.6)	31.8(25.9–38.3)	18.0(13.4–23.8)
	*p* value	<0.001	0.080	0.078
Family history of coronary heart disease	Yes	17.6(14.5–21.2)	43.0(33.0–53.6)	23.7(16.0–33.7)
	No	13.2(11.6–15.1)	29.6(23.5–36.5)	18.1(13.0–24.6)
	*p* value	0.018	0.030	0.283
Family history of dyslipidemia	Yes	15.0(11.8–18.9)	40.3(28.2–53.9)	17.5(9.7–29.5)
	No	14.2(12.5–16.0)	31.7(26.0–37.9)	20.7(15.7–26.8)
	*p* value	0.683	0.224	0.595

#### Awareness of dyslipidemia

The awareness of dyslipidemia generally increased with increasing age (*p* = 0.007), with the last age group 70– having the highest level of awareness, 18.9% (95% CI: 13.3–26.0%); additionally, those living in the urban areas, with a high educational level, and those who were lack of exercise, overweight or obesity, with personal history of stroke or coronary heart disease, and family history of coronary heart disease usually had a higher rate of awareness than those living in the rural areas, with a low educational level, and those who got enough exercise, had a relatively normal body mass index, without personal history of stroke or coronary heart disease, or family history of coronary heart disease (all *p* < 0.05). However, the awareness of dyslipidemia was not significantly associated with gender (*p* = 0.287), smoking (*p* = 0.236) or alcohol consumption status (*p* = 0.185).

#### Treatment of dyslipidemia

Among participants who were aware of dyslipidemia, those with family history of coronary heart disease were more likely to treat their dyslipidemia than those without family history of coronary heart disease (*p* = 0.030). In the present study, treatment of dyslipidemia was not found to be significantly associated with age (*p* = 0.763), gender (*p* = 0.320), residential area (*p* = 0.237), education level (*p* = 0.225), exercise habits (*p* = 0.128), smoking (*p* = 0.245) or alcohol consumption status (*p* = 0.282), BMI (*p* = 0.476), personal history of stroke (*p* = 0.147) or coronary heart disease (*p* = 0.080), or family history of stroke (*p* = 0.122) or dyslipidemia (*p* = 0.224).

#### Control of dyslipidemia

Among dyslipidemia patients using lipid-lowering drugs, participants with regular exercise were more likely to have their dyslipidemia controlled than those who were lack of exercise (23.4% vs. 10.2%, *p* = 0.013). Besides, overweight or obesity (BMI ≥ 26 kg/m^2^) was associated with a lower level of dyslipidemia control than those with BMI <26 kg/m^2^ (*p* = 0.006).

### Factors associated with the prevalence, awareness, treatment, and control of dyslipidemia

Multiple logistic regression analysis (Table 4) suggests that the prevalence of dyslipidemia was significantly associated with increasing age; and participants living in urban regions (OR = 1.339; 95% CI: 1.161, 1.543) were more inclined to suffer from dyslipidemia. Overweight or obesity (OR = 2.156; 95% CI: 1.863, 2.533) and lack of exercise (OR = 1.212; 95% CI: 1.021, 1.439) were associated with an increased prevalence of dyslipidemia. Subjects with hypertension (OR = 1.643; 95% CI: 1.425, 1.893), or diabetes mellitus (OR = 2.173; 95% CI: 1.661, 2.844) were more likely to have dyslipidemia. In addition, participants with personal (OR = 1.365; 95% CI: 1.006, 1.851) or family history of coronary heart disease (OR = 1.182; 95% CI: 1.004, 1.391), or family history of dyslipidemia (OR = 2.135; 95% CI: 1.710, 2.666) were more likely to have dyslipidemia compared to those without any history of these conditions.

**Table 4:** Multivariate logistic regression analyses on influence factors for prevalence of dyslipidemia

Category	Subcategory	Fully adjusted OR (95% CI)	Wald *χ* ^2^ value	*p* value
Age(years)	40–49	1.00 (Referent)		
	50–59	1.506(1.277–1.776)	23.739	<0.001
	60–69	1.524(1.267–1.834)	19.933	<0.001
	70–	1.335 (1.006–1.772)	3.994	0.046
Gender	Female	1.00 (Referent)		
	Male	0.914(0.795–1.052)	1.567	0.211
Residence	Rural	1.00 (Referent)		
	Urban	1.339(1.161–1.543)	16.184	<0.001
Overweight or obesity	No	1.00 (Referent)		
	Yes	2.156(1.836–2.533)	87.602	<0.001
Lack of exercise	No	1.00 (Referent)		
	Yes	1.212(1.021–1.439)	4.810	0.028
Hypertension	No	1.00 (Referent)		
	Have	1.643(1.425–1.893)	47.023	<0.001
Diabetes mellitus	No	1.00 (Referent)		
	Have	2.173(1.661–2.844)	32.006	<0.001
Personal history of coronary heart disease	No	1.00 (Referent)		
	Yes	1.365(1.006–1.851)	3.989	0.046
Family history of coronary heart disease	No	1.00 (Referent)		
	Yes	1.182(1.004–1.391)	4.052	0.044
Family history of dyslipidemia	No	1.00 (Referent)		
	Have	2.135(1.710–2.666)	44.886	<0.001

Method: Enter: Age, Gender, Residence; Forward-Conditional: Overweight or obesity, Hypertension, Diabetes mellitus, Lack of exercise, Personal history of stroke, Personal history of coronary heart disease, Family history of coronary heart disease, Family history of dyslipidemia

*Abbreviations*: *OR* odds ratio, *CI* confidence interval

As shown in Table 5, participants living in urban areas (OR = 1.368; 95% CI: 1.021, 1.834) were more likely to be aware of their dyslipidemia condition than those living in the rural areas. Subjects with education level of senior middle school (OR = 1.963; 95% CI: 1.305, 2.953) or college and above (OR = 2.325; 95% CI: 1.447, 3.734) tended to be more aware of their dyslipidemia condition than those with education level of primary school and below. Participants overweight or obesity (OR = 1.881; 95% CI: 1.499, 2.359), or those with hypertension (OR = 1.751; 95% CI: 1.339, 2.289) or diabetes mellitus (OR = 1.727; 95% CI: 1.305, 2.286) were more inclined to be aware of their dyslipidemia condition. In addition, having a personal history of coronary heart disease (OR = 2.595; 95% CI: 1.864, 3.613) or stroke (OR = 2.176; 95% CI: 1.552, 3.051) increased the tendency of dyslipidemia awareness.

**Table 5:** Multivariate logistic regression analyses on influence factors for awareness of dyslipidemia

Category	Subcategory	Fully adjusted OR (95% CI)	Wald *χ* ^2^ value	*p* value
Age(years)	40–49	1.00 (Referent)		
	50–59	1.133(0.848–1.515)	0.712	0.399
	60–69	1.403(1.023–1.924)	4.419	0.036
	70–	1.221 (0.770–1.938)	0.719	0.396
Gender	Female	1.00 (Referent)		
	Male	1.011(0.802–1.273)	0.008	0.928
Residence	Rural	1.00 (Referent)		
	Urban	1.368(1.021–1.834)	4.398	0.036
Education	Primary school and below	1.00 (Referent)		
	Junior middle school	1.252(0.916–1.713)	1.987	0.159
	Senior middle school	1.963(1.305–2.953)	10.487	0.001
	College and above	2.325(1.447–3.734)	12.162	<0.001
Overweight or obesity	No	1.00 (Referent)		
	Yes	1.881(1.499–2.359)	29.850	<0.001
Hypertension	No	1.00 (Referent)		
	Have	1.751(1.339–2.289)	16.787	<0.001
Fruit consumption	≤2d/w	1.00 (Referent)		
	3-4d/w	0.883(0.445–1.753)	0.127	0.722
	≥5d/w	0.319(0.134–0.759)	6.678	0.010
Diabetes mellitus	No	1.00 (Referent)		
	Have	1.727(1.305–2.286)	14.621	<0.001
Personal history of coronary heart disease	No	1.00 (Referent)		
	Yes	2.595(1.864–3.613)	31.878	<0.001
Personal history of stroke	No	1.00 (Referent)		
	Yes	2.176(1.552–3.051)	20.314	<0.001

Method: Enter: Age, Gender, Residence; Forward-Conditional: Education, Overweight or obesity, Hypertension, Diabetes mellitus, Fruit consumption, Smoking, Alcohol consumption, Lack of exercise, Personal history of stroke, Personal history of coronary heart disease, Family history of coronary heart disease, Family history of stroke

*Abbreviations*: *OR* odds ratio, *CI* confidence interval

Table 6 indicates that subjects with a personal history of coronary heart disease (OR = 2.021; 95% CI: 1.183, 3.453) or stroke (OR = 1.743; 95% CI: 1.002, 3.032) were more inclined to receive treatment for dyslipidemia than those without these conditions.

**Table 6:** Multivariate logistic regression analyses on influence factors for treatment of dyslipidemia

Category	Subcategory	Fully adjusted OR (95% CI)	Wald *χ* ^2^ value	*p* value
Age(years)	40–49	1.00 (Referent)		
	50–59	1.098(0.627–1.922)	0.107	0.744
	60–69	0.892(0.492–1.615)	0.143	0.705
	70–	1.134(0.494–2.601)	0.088	0.767
Gender	Female	1.00 (Referent)		
	Male	0.769(0.500–1.182)	1.435	0.231
Residence	Rural	1.00 (Referent)		
	Urban	0.827(0.537–1.272)	0.750	0.386
Personal history of coronary heart disease	No	1.00 (Referent)		
	Yes	2.021(1.183–3.453)	6.634	0.010
Personal history of stroke	No	1.00 (Referent)		
	Yes	1.743(1.002–3.032)	3.873	0.049

Method: Enter: Age, Gender, Residence; Forward-Conditional: Personal history of stroke, Personal history of coronary heart disease, Family history of coronary heart disease

*Abbreviations*: *OR* odds ratio, *CI* confidence interval

Table 7 illustrates that overweight or obesity (OR = 0.404; 95% CI: 0.235, 0.695) and lack of exercise (OR = 0.423; 95% CI: 0.215, 0.830) were associated with poor control of dyslipidemia; and subjects with personal history of coronary heart disease (OR = 2.065; 95% CI: 1.083, 3.905) were more likely to control their serum lipids at normal levels.

**Table 7:** Multivariate logistic regression analyses on influence factors for control of dyslipidemia

Category	Subcategory	Fully adjusted OR (95% CI)	Wald *χ* ^2^ value	*p* value
Age(years)	40–49	1.00 (Referent)		
	50–59	0.544(0.264–1.123)	2.707	0.100
	60–69	0.830(0.408–1.687)	0.266	0.606
	70–	1.174(0.458–3.013)	0.112	0.738
Gender	Female	1.00 (Referent)		
	Male	1.345(0.787–2.299)	1.178	0.278
Residence	Rural	1.00 (Referent)		
	Urban	0.640(0.376–1.091)	2.692	0.101
Overweight or obesity	No	1.00 (Referent)		
	Yes	0.404(0.235–0.695)	10.720	0.001
Lack of exercise	No	1.00 (Referent)		
	Yes	0.423(0.215–0.830)	6.264	0.012
Personal history of coronary heart disease	No	1.00 (Referent)		
	Yes	2.056(1.083–3.905)	4.852	0.028

Method: Enter: Age, Gender, Residence; Forward-Conditional: Overweight or obesity, Diabetes mellitus, Fruit consumption, Lack of exercise, Personal history of coronary heart disease, Family history of coronary heart disease

*Abbreviations*: *OR* odds ratio, *CI* confidence interval

## Discussion

In this population-based cross-sectional epidemiological study, we have identified a high prevalence of dyslipidemia among adults aged 40 years and over in northeast China, especially in urban areas. Additionally, we found that high triglyceride was the most prevalent type of dyslipidemia in northeast China, followed by high total cholesterol. However, the awareness, treatment, and control rate of dyslipidemia were at far from desirable levels. Increasing age, living in urban regions and family history of dyslipidemia were associated with higher risk of dyslipidemia. But beyond that, participants with underlying chronic diseases, such as overweight or obesity, hypertension, or diabetes mellitus were at a higher risk of dyslipidemia; also these people were more likely to be aware of their condition. However, this did not increase the likelihood of treatment and control of dyslipidemia. Subjects living in urban areas and having higher educational level tended to be more aware of their dyslipidemia condition. Our study also suggests that subjects with personal history of coronary heart disease were most likely to be aware of, treat, and control their dyslipidemia condition; also subjects with personal history of stroke were more likely to be aware of and treat their dyslipidemia condition, but it didn’t mean well control of their dyslipidemia. Overweight or obesity and lack of exercise were also associated with poor dyslipidemia control.

The first national study on serum lipids was part of the China National Nutrition and Health Survey performed in 2002 by China’s Center for Disease Control and Prevention [17], which showed that the prevalence of dyslipidemia in Chinese adults aged 18 and over was 18.6%, with 17.0, 22.9, and 23.4% in the age groups of 18–44, 45–59, and over 60 years old, respectively. In this representative sample of northeast Chinese adults aged 40 years and over of our study, the overall prevalence of dyslipidemia was 62.1%, with 58.6%, 64.4%, 65.7%, 63.3% in the age groups of 40–49, 50–59, 60–69, and over 70 years old, respectively. These prevalence rates in each age group over 40 years were significantly higher than data reported by the China National Survey of Chronic Kidney Disease Working Group, conducted from January 2007 to October 2010 [18, 19]. The cross-sectional survey covered 13 provinces but did not contain any provinces in northeast China, reporting that the adjusted prevalence of dyslipidemia in Chinese adults in the age groups of 40–49, 50–59, 60–69, and over 70 years old was 43.6, 46.8, 45.3, and 42.9%, respectively, for males, and 23.5, 39.9, 50.0, and 46.6%, respectively, for females. Our study also showed that high triglyceride was the most prevalent form of dyslipidemia in northeast Chinese adults aged 40 years and over, followed by high total cholesterol (the prevalence of high total cholesterol, triglyceride, low-density lipoprotein cholesterol, and low high-density lipoprotein cholesterol were 33.5, 43.9, 0.6, and 8.8%, respectively). This was consistent with the overall pooled analysis, including thirty-eight studies on the epidemiology of dyslipidemia in Chinese adults [13]; however, this was different from many other national cross-sectional studies [18, 20, 21], in which low high-density lipoprotein cholesterol or high low-density lipoprotein cholesterol was the major type of dyslipidemia. In addition to the differences in methodology and population demography, local climate conditions, dietary habits, and sedentary lifestyles might contribute to the high prevalence of dyslipidemia [22], especially high triglyceride and high total cholesterol in the northeast Chinese adults aged 40 years and over. Jilin Province, located in the center of northeast China, has a temperate continental monsoon climate. The cold climate leads to a deficiency of fresh fruits and vegetables; thus, people eat relatively more animal fat and fewer fresh vegetables and fruits. This diet leads to a high prevalence of high triglyceride and total cholesterol. Moreover, the cold weather limits people’s outdoor physical activity during the long winter, consequently increasing the risk of overweight or obesity and related metabolic abnormalities. Elevated triglycerides [10] and high total cholesterol levels [8], as predictive of cardiovascular risk, have been well-established. Therefore, lowering triglyceride and total cholesterol levels is the primary and urgent target for cardiovascular disease prevention in northeast China.

In the present study, factors associated with the prevalence of dyslipidemia included increasing age, living in urban regions, having personal or family history of coronary heart disease, and family history of dyslipidemia. Our study also found that overweight or obesity and lack of exercise were associated with an increased the risk of dyslipidemia. In addition, people with underlying chronic diseases, such as hypertension, or diabetes mellitus were at a higher risk of dyslipidemia. These results were partly consistent with many previous studies [18, 20, 21, 23]. It may reflect that factors associated with dyslipidemia and the consequences of dyslipidemia (such as overweight or obesity) are similar among different ethnicities. This suggests that people with underlying chronic diseases, such as obesity, hypertension, and diabetes mellitus are at a higher risk of developing dyslipidemia and should be more carefully monitored and managed.

Dyslipidemia is one of the most important independent modifiable risk factors for cardiovascular disease in both Western [24, 25] and Asian [26, 27] populations. Raising the awareness, treatment and control rate of dyslipidemia has a positive impact on the primary and secondary prevention of cardiovascular disease. In 2010, Li et al. [28] reported that the awareness rate of dyslipidemia among Chinese adults aged 45–59 and over 60 years old was 16.75 and 18.74%, respectively. The treatment rate was 10.73 and 12.05% in the corresponding age groups, and the control rates were was 5.49 and 6.94%, respectively. Our study showed that the awareness, treatment, and control rate of dyslipidemia in northeast Chinese adults aged 40 years and over were 14.4, 33.9, and 19.9%, respectively. These rates were significantly higher than the China National Diabetes and Metabolic Disorders Study [29] conducted from June 2007 to May 2008, in which the awareness, treatment, and control of borderline high or high total cholesterol was 11.0, 5.1, and 2.8%, respectively. This showed that with the great importance attached to early screening, health education, and intervention therapy in recent years, people’s awareness and prevention methods for chronic disease are being improved. Even so, these rates are still far below those from Canada and the US [30]. In the current study, we also found that lipid-lowering drugs alone would not solve the problem of dyslipidemia control completely. Even in those who were receiving treatment for dyslipidemia, only 19.9% participants control their serum lipids at normal levels. In addition to increasing rates of statin prescription and lifestyle intervention in the patients with severe dyslipidemia [31–33], we should popularize the awareness of nutraceuticals and functional food ingredients in influencing dyslipidemia in the public. Scicchitano P et al.[34] suggested that nutraceutical and functional food ingredients, such as resveratrol, polyphenols, proanthocyanidins, fish oil, etc., can be adopted in the common pharmacological treatments for dyslipidemia, such as statin therapy. They can positively influence lipid profile by combining the effects of drug therapy. What’s more, they can be considered as an alternative therapeutic option in situations where statins cannot be used because of intolerance.

In this study, we found that subjects with a personal history of coronary heart disease or stroke were more likely to be aware of and treat their dyslipidemia condition. Moreover, subjects with personal history of coronary heart disease were more inclined to control their serum lipids at normal levels, while this was not found in the subjects with personal history of stroke. Previous researches [35–37] have shown that those who had a cardiovascular event before often become more focused on their health, being particularly concerned about cardiovascular disease risk factors, including dyslipidemia, and these patients always were more probably to engage in and comply with lipid-lowering drugs or lifestyle intervention for dyslipidemia. Our study also revealed that those highly educated usually had a higher rate of awareness than those with low education levels. This finding was consistent with several other studies [14, 38–41]. Previous report has suggested that education is the best socioeconomic status index and predicts awareness of risk factors for cardiovascular disease [42]. The higher the level of education attained by an individual, the more likely they are to express increased awareness about health conditions, including dyslipidemia.

This population-based cross-sectional epidemiologic study was conducted in a large representative sample of northeast Chinese adults aged 40 and over. The data were taken from the 2015–16 Stroke Screening and Prevention Program in northeast China. Standard protocols and instruments designed by the Stroke Screening and Prevention Program of the National Health and Family Planning Commission of China, along with strict training processes for data collection, were used to ensure the high quality of the data. In addition, standard laboratory methods for the measurement of serum lipids were used, and the sample was uniformly measured by Changchun Kingmed Center for Clinical Laboratory Co., Ltd. For all the aforementioned reasons, our study provides the most reliable and up-to-date information on the prevalence, awareness, treatment, and control of dyslipidemia in adults aged 40 and over in northeast China.

However, the findings of our present study should be interpreted with an understanding of the following limitations. First, as these findings were derived from a cross-sectional study without a strict follow-up design, and the prevalence, awareness, treatment, and control of dyslipidemia were based on questionnaire and measurements taken by a single visit, the results are prone to be affected by the recall bias and unmeasured confounding. Second, the definition of dyslipidemia in our study was based on Chinese guidelines on prevention and treatment of dyslipidemia in adults; therefore, the comparison between our results and those from other countries should be carefully made. Finally, no causal relationships could be precisely delineated for the properties of the cross-sectional study.

## Conclusions

In conclusion, we have identified a high prevalence of dyslipidemia among adults aged 40 years and over in northeast China, especially in the urban areas. Our cross-sectional study showed that high triglycerides and high total cholesterol are two major types of dyslipidemia in northeast China. However, the awareness, treatment, and control rates of dyslipidemia are far from the desirable levels. Renewed efforts taking influence factors into account are needed to lower the prevalence of dyslipidemia and increase the awareness, treatment, and control rates of dyslipidemia. These efforts includes strengthening the primary care system, promotion of a healthy lifestyle, and energetically improve people’s educational degrees.

## Abbreviations

BMI: Body mass index
CI: Confidence interval
DBP: Diastolic blood pressure
FBG: Fasting blood-glucose
HDL-C: High-density lipoprotein cholesterol
H-LDL: High low-density lipoprotein cholesterol
H-TC: High total cholesterol
H-TG: High triglycerides
LDL-C: Low-density lipoprotein cholesterol
L-HDL: Low high-density lipoprotein cholesterol
OR: Odds ratio
SBP: Systolic blood pressure
TC: Total cholesterol
TGs: Triglycerides
